# Risk Of Human Dietary Exposure To Organochlorine Pesticide Residues In Fruits From Ghana

**DOI:** 10.1038/s41598-018-35205-w

**Published:** 2018-11-12

**Authors:** Frederick Forkuoh, Nathaniel Owusu Boadi, Lawrence Sheringham Borquaye, Samuel Afful

**Affiliations:** 1 Department of Chemistry, Kwame Nkrumah University of Science and Technology, Kumasi, Ghana; 2 Nuclear Chemistry Environmental Research Center, Ghana Atomic Energy Commission, Box LG. 80, Legon, Accra Ghana

## Abstract

The objectives of this present study were to assess the level of organochlorine pesticide (OCP) residues in fruits and to determine the potential health risks associated with the exposure to these pesticides. A total of 120 fruits (watermelon, pineapple, and banana) were collected from five communities and a local market within the Mampong Municipality and analyzed for organochlorine pesticide residues. The results showed that the concentrations ranged from not detectable (ND)–48.22 ng/g for DDTs, ND–19.03 ng/g for HCHs, ND–4.10 ng/g for CHLs, ND–22.84 ng/g for Aldrin, and ND–11.53 ng/g for other OCPs. Levels of methoxychlor, Aldrin and gamma-hexachlorocyclohexane (HCH) exceeded the maximum residue limits in watermelon. Estimated health risk revealed that Aldrin in watermelon could pose potential toxicity to the consumer. Estimated average daily intake for Aldrin was above the acceptable average daily intake.

## Introduction

Organochlorine pesticides have been reduced significantly in developed countries due to their ban, however, they are still being used in many developing countries^1–3^. Organochlorine pesticides are stable and stay in the environment for a long time. Pesticides such as hexachlorocyclohexane (HCH), endosulfan and DDT have been used for agricultural purposes^4–7^. The most dominant OCP residues in the environment are DDTs and HCHs^8^.

Organochlorine pesticide residues have the tendency to enter the tissues of plants. These residues have been found in the fruit pulp and juice^9^. In order to ensure the health of consumers and manage the efficient use of agricultural resources as well as make some economic gains, the National food programmes were established globally^9–12^.

Ghana’s food needs keep increasing due to a continuous increase in population. This is not different from other nations in the world. Fruits are an integral part of our food needs to ensure balanced diet. Pesticides are used in the cultivation of these fruits to prevent pests from destroying the plants and boost production. Fruits are the sources of most vitamins for human nutrition and improves vision^13^. Fruits also contain antioxidants which help prevent diseases such as diabetes mellitus, cancer, cardiac disorders, inflammations and neurodegenerative diseases^14^.

Ghana used to cultivate fruits on subsistence basis but this has advanced to commercial levels and Ghana currently exports fruits such as pineapple and banana to other countries^15^. The major challenge with fruit cultivation is the variety of pests and diseases that attack the plants. Pest control practices in Ghana in fruit production include highly toxic pesticide applications that are usually misapplied, leading to pesticide contamination of the products and the environment.

In Ghana, OCP residues have been detected in agricultural products produced and consumed^16–18^. Moreover, there have been a lot of reports worldwide about pesticide residues in grains^11^, vegetables^19^, milk^20^ and fish^21^. Studies conducted within the Kumasi Metropolis recently on the levels of OCP residues in vegetables within the Kumasi Metropolis indicated potential usage of OCP s currently, despite its ban^22^. However, data on OCP residues in fruits grown in the Mampong municipality is lacking. This study evaluates the residual concentrations of OCPs in selected fruits produced within the Mampong Municipality and evaluate the human health impact associated with the consumption of these fruits. The results indicate that consumers, both adults and children are at risk of consumption.

## Methods and Materials

**Figure 1:**
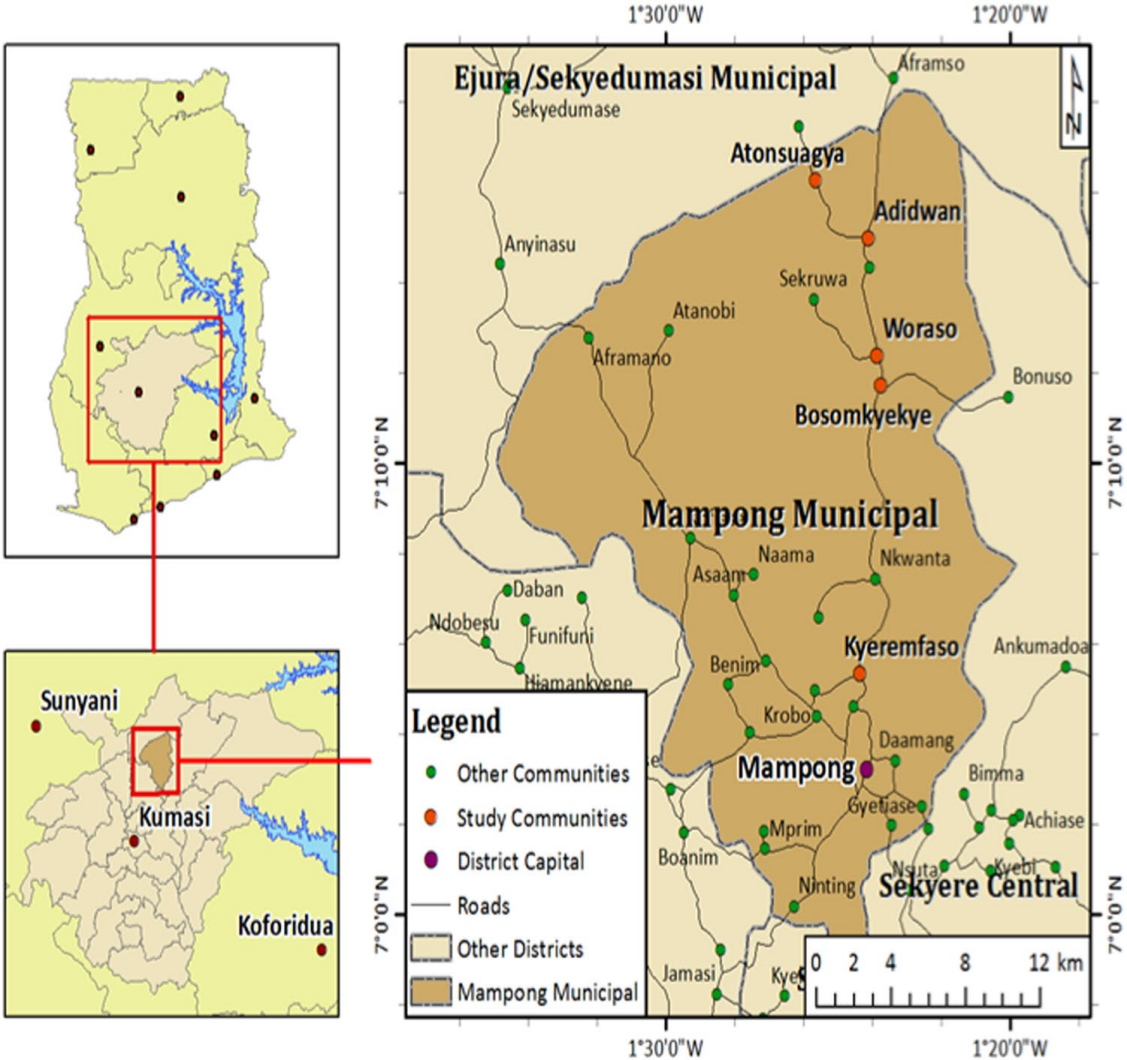
Map of the study area showing the sampling points.

The study was conducted at Adidwan, Woraso, Bosomkyekye, Atonsuagya and kyerefamso, all in the Mampong municipality. Mampong municipality is located in the northeast of Kumasi, the Ashanti regional capital. A map of the study area is shown in Fig. 1.

A total of 100 samples of freshly harvested fruits were collected from farms in Kyerefamso, Woraso, Adidwan, Atonsuagya and Bosomkyekye in the Mampong Municipality. The sampling points are shown in Fig. 1. The farms were selected based on the farmers’ active use of pesticides. The samples collected were then wrapped in aluminium foil and sealed in polyethene bags, labelled and transferred to the laboratory. They were then stored in a refrigerator prior to sample preparation and analysis.

All glassware for the extraction and clean-up were initially washed with soap and tap water and rinsed several times with distilled water. The glassware were then further rinsed with acetone and oven-dried at 60 °C. Organochlorine pesticide standard for Gas Chromatography was purchased from Dr Ehrenstorfer Laboratories (GmbH Germany). The standard was prepared in acetone and stored at −20 °C. Serial dilutions were prepared from the standard stock in acetone for analysis. Ethyl acetate, hexane, diethyl ether and acetone were obtained from BDH Laboratory Supplies, England. Analytical grades of sodium sulphate and sodium hydrogen carbonate were also purchased from BDH Laboratory Supplies, England. Florisil and silica adsorbents were purchased from Hopkins and William Limited, England.

The method adopted by Bempah *et al*.^17^ was used for the extraction of fruit samples. Samples of fresh fruits were washed several times with distilled water, peeled and chopped with a stainless steel knife and homogenized using a blender. About 20.0 g of the homogenized sample was macerated with 40 mL of ethyl acetate. Approximately 5.0 g Sodium bicarbonate and 20.0 g anhydrous sodium sulphate were added to dry the extract and the sample was further macerated for 3 minutes. The macerated samples were then centrifuged for 5 minutes at 3000 rpm to obtain the two phases. The supernatants were transferred to pre-cleaned Pyrex beakers.

The clean-up procedure was performed according to the method of Kuranchie-Mensah *et al*.^23^. Combined florisil- silica solid phase extraction columns were prepared by packing 1.5 and 0.5 g of pre-activated florisil and silica, respectively with 1.0 g of sodium sulphate placed above the adsorbents in a glass column. The columns were then conditioned with 10 mL of hexane before clean up. The extract was passed through the column and the eluate collected in a 50 mL conical flask. The columns were first eluted with 15 mL of hexane, then 5 mL of 2:1 hexane/diethyl ether mixture. The eluate was concentrated almost to dryness on a rotary evaporator and taken up in 1.5 mL of ethyl acetate. The extract was finally transferred into 2 mL glass vial using a Pasteur pipette for GC - ECD analysis.

Organochlorine residues were determined using a Varian CP-3800 gas chromatograph with ^63^Ni electron capture detector (GC-ECD) and VF-5ms capillary column (40 m × 0.25 mm × 0.25 µm). The injector temperature was operated at 270 °C with an injection volume of 1 µL. Oven temperature was programmed as follows: the initial temperature was 80 °C (holding for 1 min), which was increased to 300 °C at a rate of 25 °C/min (holding for 1 min). Nitrogen was used as carrier gas flowing at the rate of 30 mL/min. The temperature for the detector was set at 300 °C. Levels of organochlorine pesticides were recognized based on the comparison of retention times with known standards and quantitated by the method of external standard.

Quality assurance and quality control were included in the analytical scheme. Quality of pesticide residue analysis was established through solvent blanks analysis, spikes and triplicate samples. Solvent blanks were used to eliminate the interference of the reactants, while the spike samples were used for determination of recovery. Triplicate samples were used to confirm the accuracy of the method. The spiked samples and blank were subjected to the same extraction and clean up performed on each sample followed by GC analysis and quantification. Recoveries ranged from 76.93 to 112% of organochlorine pesticide residues.

The integrated statistical analysis in the work included means and standard deviations equivalents. Data were also analyzed for ANOVA using IBM SPSS ’20.0 to establish the differences in pesticide residues between samples, as well as the different sampling sites. All tests were considered statistically significant at p < 0.05.

To evaluate the potential health risk associated with each OCP residues, certain assumptions based on guidelines from the Environmental Protection Agency^24^ were taken into considerations. It was assumed that the theoretical body weight of a child is 10 kg and that of an adult is 60 kg. It was also assumed that absorption and bioavailability rates are 100%. Fruit consumption rate in Ghana is estimated at 64 g/day^17^. The estimated daily intake (ng/g) of the individual organochlorine pesticide was calculated by multiplying the average pesticide concentrations (ng/g) in the fruit of the interest by the fruit consumption rate (g/day) and dividing the product by the body weight (g).

1 $${\rm{ED}}=\frac{{\rm{Cp}}\times {\rm{FCR}}}{{\rm{BW}}}$$

ED is estimated dose, Cp is the pesticide concentration (ng/g), FCR is defined as the food consumption rate (g/day) and BW is the average body weight (g) of children and adults in Ghana.

Risk of organochlorine pesticides to children and adults through daily fruit consumption was classified on the basis of the guidelines proposed by the US EPA^24^. For non-cancer effects, hazard indices (HI) for adults and children were calculated as the ratio of estimated dose (ED) to reference dose of the pesticides.

2 $${\rm{HI}}=\frac{{\rm{ED}}}{{{\rm{R}}}_{{\rm{f}}}{\rm{D}}}$$

Where ED is the estimated dose, and R_f_D is the reference dose. The exposure to multiple pesticides may result in commutative effects. The method of risk index established by the EPA was used to assess risks to the health of humans by a group of pesticides that are similar toxicologically. The cumulative hazard index (HI) was evaluated using the equation below^25^.

3 $${\rm{HI}}=\frac{{{\rm{ED}}}_{1}}{{{\rm{R}}}_{{\rm{f}}}{{\rm{D}}}_{1}}+\frac{{{\rm{ED}}}_{2}}{{{\rm{R}}}_{{\rm{f}}}{{\rm{D}}}_{2}}+\ldots +\frac{{{\rm{ED}}}_{{\rm{n}}}}{{{\rm{R}}}_{{\rm{f}}}{{\rm{D}}}_{{\rm{n}}}}={\sum }_{{\rm{i}}=1}^{{\rm{n}}}\frac{{{\rm{ED}}}_{{\rm{i}}}}{{{\rm{R}}}_{{\rm{f}}}{{\rm{D}}}_{1}}$$

Where ED_1_, ED_2_, ED_n_ and ED_i_ are the estimated dose of each individual pesticide. R_f_D_1_, R_f_D_2_, R_f_D_n_ and R_f_D_i_ are the reference doses for each pesticide.

Carcinogenic risks related to the exposure to OCP residues in fruits were evaluated. The benchmark concentration of carcinogenic effect was estimated using US EPA oral slope factor^26^. Risk assessments were analyzed on the basis of OCP residue levels in the fruit. Risk ratios (HR) were determined by dividing the estimated dose (ED) by the reference concentrations (CBC)^27^. When the hazard ratio is greater than one (1), it denotes that the estimated daily intake of the pesticide through the fruit consumption exceeds the reference concentration^27^.

4 $${\rm{Hazard}}\,{\rm{Ratio}}({\rm{HR}})=\frac{{\rm{Estimated}}\,{\rm{Dose}}({\rm{ED}})}{{\rm{Benchmark}}\,{\rm{Concentration}}({\rm{CBC}})}$$

5 $${\rm{Benchmark}}\,{\rm{Concentration}}({\rm{BC}})=\frac{{\rm{Risk}}\times {\rm{Body}}\,{\rm{weight}}}{{\rm{Fruit}}\,{\rm{Consumption}}\,{\rm{Rate}}\times {\rm{Oral}}\,{\rm{Slope}}\,{\rm{Factor}}}$$

where the risk is the probability of getting cancer throughout the lifetime due to the exposure to pesticides (1 × 10^−6^)^28^, and slope factor is cancer slope factor from EPA Integrated Risk Information (IRIS)^29^

## Results and Discussion

The combined and carcinogenic health risks associated with organochlorine pesticide residues in the fruit samples analyzed are summarized in Tables 1 and 2. The highest estimated daily dose (ED) was recorded for Aldrin in watermelon. The ED of Aldrin in watermelon for children (0.1462 ng/g) was higher than the ED for an adult (0.0244 ng/g). The estimated dose of Aldrin through the consumption of watermelon by children was higher than the reference dose (R_f_D), suggesting a potential adverse health effect on children through dietary exposure of Aldrin. The health risk from the study supports the findings that the estimated exposure levels are age-dependent and therefore children have higher food consumption per kilogram of their body weight and as a consequence have higher estimated exposure levels^30,31^. The hazard indices showed that apart from Aldrin, organochlorine pesticide residues did not show any health risk associated with the fruits.

**Table 1:** Combined risk of multiple OCPs in the fruit from the various farms.

	Watermelon		Pineapple		Banana		Total HI Children	Total HI Adult
	Children	Adult	Children	Adult	Children	Adult		
**Hazard Index (HI)**								
Adidwan	—	—	7.66 × 10^−3^	1.28 × 10^−3^	1.36 × 10^−2^	2.27 × 10^−3^	2.13 × 10^−2^	3.55 × 10^−3^
Atonsuagya	1.88 × 10^−2^	3.13 × 10^−2^	2.56 × 10^−3^	4.27 × 10^−4^	1.25 × 10^−2^	2.08 × 10^−5^	1.90 × 10^−1^	3.17 × 10^−3^
Bosomkyekye	7.28 × 10^−2^	1.21 × 10^−2^	8.28 × 10^−3^	1.38 × 10^−3^	3.78 × 10^−4^	6.29 × 10^−5^	8.15 × 10^−2^	1.36 × 10^−2^
Kyerefamso	2.71	4.52 × 10^−2^	8.29 × 10^−4^	1.38 × 10^−4^	6.86 × 10^−1^	1.14 × 10^−1^	3.40	5.67 × 10^−1^
Woraso	—	—	—	—	—	—	—	—
All farms	2.97	4.96 × 10^−2^	1.93 × 10^−2^	3.22 × 10^−3^	7.00 × 10^−1^	1.17 × 10^−1^	3.69	6.16 × 10^−1^

**Table 2:** Carcinogenic risks for OCP residues in watermelon from all farms.

Pesticide	Watermelon		Pineapple		Banana	
	Children	Adult	Children	Adult	Children	Adult
	HR	HR	HR	HR	HR	HR
Gamma - HCH	4.11 × 10^−2^	1.14 × 10^−2^	1.18 × 10^−1^	3.27 × 10^−3^	1.86 × 10^−2^	5.16 × 10^−4^
Beta – HCH	8.72 × 10^−2^	2.42 × 10^−2^	—	—	1.48 × 10^−1^	5.62 × 10^−3^
Heptachlor	3.73 × 10^−2^	1.04 × 10^−3^	—	—	—	—
Delta - HCH		—	—	—	—	—
Aldrin	5.51 × 10^−2^	1.53 × 10^−3^	—	—	1.04 × 10^−2^	2.89 × 10^−4^
Gamma- chlordane	—	—	—	—	—	—
A - endosulfan	—	—	—	—	—	—
p,p’ – DDE	—	—	—	—	—	—
Dieldrin	2.2 × 10^−2^	6.11 × 10^−4^	—	—	—	—
Endrin	—	—		—	—	—
p,p’ – DDT	2.48	6.89 × 10^−2^	3.12 × 10^−1^	8.66 × 10^−3^	9.39 × 10^−2^	2.61 × 10^−3^
B - endosulfan	—	—	—	—	—	—
p,p’ – DDD	1.35 × 10^−1^	3.75 × 10^−3^	—	—	—	—
Endosulfan-s	—	—	—	—	—	—
Methoxychlor	—	—	—	—	—	—

The combined health risk of consumption of watermelon from Kyerefamso in children was 2.71. There was, however, no risk associated with the consumption of other fruits from different towns in both adults and children. The overall potential risk for noncarcinogenic health effects indicated that children were at a higher (p < 0.05) risk (3.69) than adults (0.616), for consuming fruits from the study area within the Mampong Municipality.

Carcinogenic health risk was assessed for the organochlorine pesticides due to their potential to cause cancer. The results for the carcinogenic risks have been summarized in Table 2. Hazard ratio for p,p’-DDT indicated that its contamination in watermelon could pose potential carcinogenic effect for children since the HR was >1. The carcinogenic risk of the OCP residues in fruits, in general, was of less concern since the carcinogenic rates of all the OCPs in individual fruits except p,p’-DDT were below the threshold set by the European Union.

This study has shown that organochlorine pesticide residues were found in fruits produced from the Mampong Municipality in the Ashanti region of Ghana. The residual levels of methoxychlor, Aldrin and γ-HCH obtained in watermelon for this study exceeded the EU MRLs, however, the hazard risks estimated indicated no potential hazard to the consumer. The combined health risk due to consumption of each fruit variety was minimal but, the overall health risk index due to consumption of all the fruits was higher than 1, indicating potential health risk to consumers. Since fruits form an important food item, there is the need for continuous monitoring to regulate the levels of pesticide residues in fruits and the appropriate measures taken to safeguard the health of the consumer.
